# 2,7-Dimethyl-2,7-diazo­niapyrene bis­(hexa­fluoro­phosphate)

**DOI:** 10.1107/S1600536811001978

**Published:** 2011-01-29

**Authors:** Li Ang

**Affiliations:** a College of Food Science and Biotechnology, Zhejiang Gongshang University, Hangzhou 310035, People’s Republic of China

## Abstract

In the title compound, C_16_H_14_N_2_^2+^·2PF_6_^−^, the 2,7-dimethyl-2,7-diaza­pyrenium (DM-diaz) cation lies on a crystallographic twofold rotation axes. The diaz groups are nearly coplanar, with a maximum deviation of 0.008 (3) Å. In the crystal, mol­ecules are linked into a two-dimensional lamellar framework parallel to (104) through weak C—H⋯F inter­actions.

## Related literature

For general background to 2,7-disubstituted diaza­pyrenium dications, see: Ashton *et al.* (1999 ▶); Yen *et al.* (2009 ▶); Steuerman *et al.* (2004 ▶); Lilienthal *et al.* (1996 ▶); Sindelar *et al.* (2005 ▶); Lin *et al.* (2006 ▶). For related structures, see: Blake *et al.* (1997 ▶); Dinolfo *et al.* (2004 ▶). 
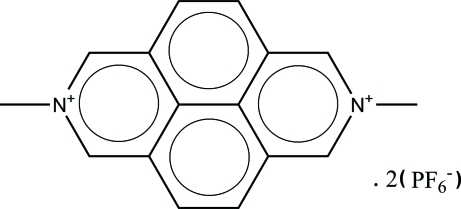


## Experimental

### 

#### Crystal data

C_16_H_14_N_2_^2+^·2PF_6_^−^
*M* *_r_* = 524.23
Monoclinic, 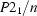

*a* = 6.7654 (14) Å
*b* = 10.653 (2) Å
*c* = 13.422 (3) Å
β = 91.03 (3)°
*V* = 967.2 (3) Å^3^
*Z* = 2
Mo *K*α radiation
μ = 0.35 mm^−1^
*T* = 293 K
0.31 × 0.31 × 0.19 mm

#### Data collection

Rigaku R-AXIS RAPID diffractometer
Absorption correction: multi-scan (*ABSCOR*; Higashi, 1995 ▶) *T*_min_ = 0.899, *T*_max_ = 0.937
7699 measured reflections
1756 independent reflections
1439 reflections with *I* > 2σ(*I*)
*R*_int_ = 0.021

#### Refinement

*R*[*F* ^2^ > 2σ(*F* ^2^)] = 0.059
*wR*(*F* ^2^) = 0.182
*S* = 1.06
1756 reflections
146 parameters
H-atom parameters constrained
Δρ_max_ = 0.48 e Å^−3^
Δρ_min_ = −0.34 e Å^−3^

### 

Data collection: *RAPID-AUTO* (Rigaku, 1998 ▶); cell refinement: *RAPID-AUTO*; data reduction: *CrystalStructure* (Rigaku/MSC, 2004 ▶); program(s) used to solve structure: *SHELXS97* (Sheldrick, 2008 ▶); program(s) used to refine structure: *SHELXL97* (Sheldrick, 2008 ▶); molecular graphics: *ORTEPII* (Johnson, 1976 ▶); software used to prepare material for publication: *SHELXL97*.

## Supplementary Material

Crystal structure: contains datablocks global, I. DOI: 10.1107/S1600536811001978/bg2385sup1.cif

Structure factors: contains datablocks I. DOI: 10.1107/S1600536811001978/bg2385Isup2.hkl

Additional supplementary materials:  crystallographic information; 3D view; checkCIF report

## Figures and Tables

**Table 1:** Hydrogen-bond geometry (Å, °)

*D*—H⋯*A*	*D*—H	H⋯*A*	*D*⋯*A*	*D*—H⋯*A*
C6—H6⋯F2^i^	0.93	2.48	3.367 (4)	160
C7—H7⋯F4^ii^	0.93	2.51	3.418 (5)	167

Symmetry codes: (i) 
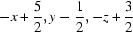
; (ii) 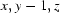.

**Fig. 1.**
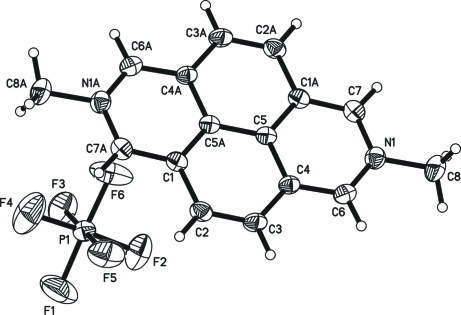
ORTEP view of the title compound. The dispalcement ellipsoids are drawn at 30% probability level. Symmetry code: (A) 2-x, 1-y, 1-z

**Fig. 2.**
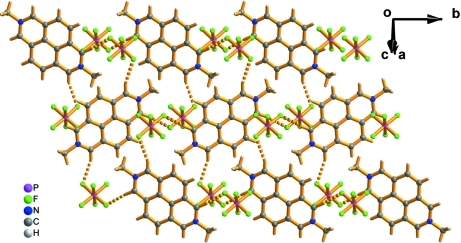
The two-dimensional layer of the compound, parallel to (101).

## supplementary crystallographic information

### Comment

2,7-Disubstituted diazapyrenium dications, which combine the features of pyrene, methylviologen, and nucletic acid intercalators, are charming pi-electron deficient building blocks in supramolecular chemistry (Ashton *et al.*, 1999; Yen *et al.*, 2009). They have been widely used as the electron-acceptors for electron-donating units such as hydroquinones and aromatic carboxylates (Steuerman *et al.*, 2004; Lilienthal *et al.*, 1996). Furthermore, due to their luminescence properties, they have also been  as fluorescence probes for ion detection (Sindelar *et al.*, 2005) and neurotransmition (Lin *et al.*, 2006). Herein, we report the crystal structure of one of these disubstituted diazapyrenium dications, the *N*,*N*'-dimethyl-2,7-diazapyrenium,  C_16_H_14_N_2_.2PF_6_, (*DM*-diaz).

The cation lies on a crystallographic twofold rotation axes; diaz groups are nearly coplanar with a maximum deviation of 0.008 (3) Å. Unlike many structures that contain diaz (Blake *et al.*, 1997; Dinolfo *et al.*, 2004), Dm-diaz exhibits no face-to-face pi-pi interactions between diaz molecules in the structure. C—H···F interactions are observed between the methyl groups of the *DM*-diaz molecules and hexafluorophoshate counterions (Table 1), forming a two-dimensional lamellar framework parallel to (101) (Figure 2).

### Experimental

A solution of 2,7-diazapyrene (0.210 g, 1.03 mmol) and iodomethane (0.568 g, 4.02 mmol) in acetonitrile (15 ml) was stirred and refluxed for 3 h. After it was cooled to room temperature, a red solid was isolated on a filter and washed with ethyl ether (30 ml). The solid was dissolved with water (75 ml) and a saturated aqueous solution of NH_4_PF_6_ (2.44 g, 15.0 mmol) was added until no further precipitate was observed. The red solid was isolated on a filter, washed with water and dried under vacuum to afford the product (0.423 g, 78.4%). Red crystals were obtained by vapor diffusion of isopropyl ether into an acetonitrile solution over a period of 5 d. ^1^H NMR (500 MHz, CD_3_CN, 295 K) δ (p.p.m.) 9.88 (4*H*,s), 8.85 (4*H*, s), 5.14 (4*H*, t, J = 5.2 Hz), 3.45 (4*H*, m), 3.45 (2*H*, t, J = 5.5 Hz).

### Refinement

H atoms bonded to C atoms were palced in geometrically calculated positionand were refined using a riding model, with C—H_aromatic_ = 0.93 Å, *C*—H_methyl_ = 0.96 Å, and with *U*_iso_(H) = 1.2 or 1.5 *U*_eq_(C).

### Crystal data

**Table:**

C_16_H_14_N_2_^2+^·2PF_6_^−^	*F*(000) = 524
*M**_r_* = 524.23	*D*_x_ = 1.800 Mg m^−^^3^
Monoclinic, *P*2_1_/*n*	Mo *K*α radiation, λ = 0.71073 Å
Hall symbol: -P 2yn	Cell parameters from 6424 reflections
*a* = 6.7654 (14) Å	θ = 3.0–27.5°
*b* = 10.653 (2) Å	µ = 0.35 mm^−^^1^
*c* = 13.422 (3) Å	*T* = 293 K
β = 91.03 (3)°	Block, yellow
*V* = 967.2 (3) Å^3^	0.31 × 0.31 × 0.19 mm
*Z* = 2	

### Data collection

**Table:**

Rigaku R-AXIS RAPID diffractometer	1756 independent reflections
Radiation source: fine-focus sealed tube	1439 reflections with *I* > 2σ(*I*)
graphite	*R*_int_ = 0.021
Detector resolution: 0 pixels mm^-1^	θ_max_ = 25.4°, θ_min_ = 3.0°
ω scans	*h* = −8→7
Absorption correction: multi-scan (*ABSCOR*; Higashi, 1995)	*k* = −12→12
*T*_min_ = 0.899, *T*_max_ = 0.937	*l* = −16→16
7699 measured reflections	

### Refinement

**Table:**

Refinement on *F*^2^	Primary atom site location: structure-invariant direct methods
Least-squares matrix: full	Secondary atom site location: difference Fourier map
*R*[*F*^2^ > 2σ(*F*^2^)] = 0.059	Hydrogen site location: inferred from neighbouring sites
*wR*(*F*^2^) = 0.182	H-atom parameters constrained
*S* = 1.06	*w* = 1/[σ^2^(*F*_o_^2^) + (0.106*P*)^2^ + 0.7563*P*] where *P* = (*F*_o_^2^ + 2*F*_c_^2^)/3
1756 reflections	(Δ/σ)_max_ < 0.001
146 parameters	Δρ_max_ = 0.48 e Å^−^^3^
0 restraints	Δρ_min_ = −0.34 e Å^−^^3^

### Special details

**Table:**

Geometry. All e.s.d.'s (except the e.s.d. in the dihedral angle between two l.s. planes) are estimated using the full covariance matrix. The cell e.s.d.'s are taken into account individually in the estimation of e.s.d.'s in distances, angles and torsion angles; correlations between e.s.d.'s in cell parameters are only used when they are defined by crystal symmetry. An approximate (isotropic) treatment of cell e.s.d.'s is used for estimating e.s.d.'s involving l.s. planes.
Refinement. Refinement of *F*^2^ against ALL reflections. The weighted *R*-factor *wR* and goodness of fit *S* are based on *F*^2^, conventional *R*-factors *R* are based on *F*, with *F* set to zero for negative *F*^2^. The threshold expression of *F*^2^ > σ(*F*^2^) is used only for calculating *R*-factors(gt) *etc*. and is not relevant to the choice of reflections for refinement. *R*-factors based on *F*^2^ are statistically about twice as large as those based on *F*, and *R*- factors based on ALL data will be even larger.

### Fractional atomic coordinates and isotropic or equivalent isotropic displacement parameters (Å^2^)

**Table:**

	*x*	*y*	*z*	*U*_iso_*/*U*_eq_	
P1	0.73691 (12)	0.87716 (8)	0.67479 (6)	0.0486 (4)	
F1	0.7852 (5)	0.9813 (3)	0.7544 (3)	0.1277 (13)	
F2	0.7768 (4)	0.7779 (3)	0.7596 (2)	0.1015 (10)	
F3	0.5103 (3)	0.8793 (2)	0.7022 (2)	0.0842 (8)	
F4	0.7012 (5)	0.9817 (4)	0.5943 (3)	0.1329 (14)	
F5	0.9644 (3)	0.8750 (3)	0.6478 (2)	0.0953 (10)	
F6	0.6946 (4)	0.7712 (3)	0.5961 (2)	0.1071 (11)	
N1	1.1916 (4)	0.2419 (2)	0.61997 (19)	0.0458 (6)	
C1	1.0682 (4)	0.6661 (3)	0.4751 (2)	0.0399 (7)	
C2	1.2564 (4)	0.6724 (3)	0.5268 (2)	0.0459 (7)	
H2	1.3272	0.7472	0.5275	0.055*	
C3	1.3308 (4)	0.5710 (3)	0.5740 (2)	0.0463 (7)	
H3	1.4527	0.5765	0.6068	0.056*	
C4	1.2250 (4)	0.4551 (3)	0.5743 (2)	0.0390 (7)	
C5	1.0387 (4)	0.4467 (2)	0.52485 (19)	0.0360 (6)	
C6	1.2952 (4)	0.3482 (3)	0.6213 (2)	0.0459 (7)	
H6	1.4170	0.3505	0.6545	0.055*	
C7	1.0142 (4)	0.2319 (3)	0.5739 (2)	0.0448 (7)	
H7	0.9465	0.1560	0.5750	0.054*	
C8	1.2764 (7)	0.1282 (3)	0.6680 (3)	0.0680 (11)	
H8A	1.1728	0.0810	0.6984	0.102*	
H8C	1.3721	0.1526	0.7180	0.102*	
H8B	1.3392	0.0774	0.6187	0.102*	

### Atomic displacement parameters (Å^2^)

**Table:**

	*U*^11^	*U*^22^	*U*^33^	*U*^12^	*U*^13^	*U*^23^
P1	0.0456 (6)	0.0520 (6)	0.0482 (6)	−0.0031 (3)	−0.0017 (4)	−0.0009 (4)
F1	0.142 (3)	0.105 (2)	0.136 (3)	−0.007 (2)	−0.003 (2)	−0.068 (2)
F2	0.097 (2)	0.105 (2)	0.103 (2)	0.0185 (16)	−0.0030 (16)	0.0420 (17)
F3	0.0548 (14)	0.105 (2)	0.0938 (18)	0.0116 (12)	0.0153 (12)	0.0123 (14)
F4	0.109 (2)	0.150 (3)	0.140 (3)	−0.012 (2)	−0.008 (2)	0.093 (2)
F5	0.0494 (13)	0.125 (2)	0.112 (2)	−0.0215 (13)	0.0103 (13)	−0.0286 (17)
F6	0.0775 (16)	0.143 (3)	0.101 (2)	−0.0446 (17)	0.0222 (15)	−0.0672 (19)
N1	0.0497 (14)	0.0464 (14)	0.0414 (13)	0.0030 (11)	0.0029 (11)	0.0041 (11)
C1	0.0359 (14)	0.0422 (15)	0.0418 (15)	−0.0054 (12)	0.0052 (12)	−0.0036 (12)
C2	0.0366 (15)	0.0449 (17)	0.0561 (19)	−0.0096 (12)	−0.0002 (14)	−0.0050 (14)
C3	0.0322 (14)	0.0558 (18)	0.0509 (17)	−0.0075 (13)	−0.0040 (13)	−0.0064 (14)
C4	0.0323 (14)	0.0464 (16)	0.0381 (14)	−0.0014 (11)	−0.0005 (11)	−0.0040 (12)
C5	0.0325 (14)	0.0415 (15)	0.0341 (14)	−0.0027 (11)	0.0043 (11)	−0.0053 (11)
C6	0.0409 (16)	0.0567 (18)	0.0399 (16)	0.0017 (13)	−0.0031 (13)	−0.0028 (13)
C7	0.0461 (17)	0.0434 (16)	0.0451 (16)	−0.0035 (13)	0.0076 (14)	−0.0001 (13)
C8	0.078 (3)	0.057 (2)	0.068 (2)	0.0077 (18)	−0.015 (2)	0.0186 (18)

### Geometric parameters (Å, °)

**Table:**

P1—F4	1.567 (3)	C2—C3	1.346 (4)
P1—F6	1.568 (2)	C2—H2	0.9300
P1—F1	1.570 (3)	C3—C4	1.427 (4)
P1—F2	1.574 (3)	C3—H3	0.9300
P1—F3	1.583 (2)	C4—C6	1.382 (4)
P1—F5	1.587 (2)	C4—C5	1.417 (4)
N1—C6	1.332 (4)	C5—C5^i^	1.413 (5)
N1—C7	1.344 (4)	C6—H6	0.9300
N1—C8	1.483 (4)	C7—H7	0.9300
C1—C7^i^	1.382 (4)	C8—H8A	0.9600
C1—C5^i^	1.402 (4)	C8—H8C	0.9600
C1—C2	1.440 (4)	C8—H8B	0.9600
			
F4—P1—F6	91.3 (2)	C1—C2—H2	119.7
F4—P1—F1	89.7 (2)	C2—C3—C4	120.8 (3)
F6—P1—F1	178.30 (18)	C2—C3—H3	119.6
F4—P1—F2	176.9 (2)	C4—C3—H3	119.6
F6—P1—F2	91.8 (2)	C6—C4—C5	117.2 (3)
F1—P1—F2	87.2 (2)	C6—C4—C3	123.1 (3)
F4—P1—F3	90.70 (16)	C5—C4—C3	119.7 (3)
F6—P1—F3	90.07 (15)	C1^i^—C5—C5^i^	120.2 (3)
F1—P1—F3	91.28 (17)	C1^i^—C5—C4	120.6 (3)
F2—P1—F3	89.74 (15)	C5^i^—C5—C4	119.3 (3)
F4—P1—F5	89.51 (18)	N1—C6—C4	121.2 (3)
F6—P1—F5	90.11 (14)	N1—C6—H6	119.4
F1—P1—F5	88.54 (17)	C4—C6—H6	119.4
F2—P1—F5	90.04 (17)	N1—C7—C1^i^	120.4 (3)
F3—P1—F5	179.73 (16)	N1—C7—H7	119.8
C6—N1—C7	122.6 (3)	C1^i^—C7—H7	119.8
C6—N1—C8	119.3 (3)	N1—C8—H8A	109.5
C7—N1—C8	118.1 (3)	N1—C8—H8C	109.5
C7^i^—C1—C5^i^	118.0 (3)	H8A—C8—H8C	109.5
C7^i^—C1—C2	122.6 (3)	N1—C8—H8B	109.5
C5^i^—C1—C2	119.4 (3)	H8A—C8—H8B	109.5
C3—C2—C1	120.6 (3)	H8C—C8—H8B	109.5
C3—C2—H2	119.7		

Symmetry codes: (i) −*x*+2, −*y*+1, −*z*+1.

### Hydrogen-bond geometry (Å, °)

**Table:**

*D*—H···*A*	*D*—H	H···*A*	*D*···*A*	*D*—H···*A*
C6—H6···F2^ii^	0.93	2.48	3.367 (4)	160
C7—H7···F4^iii^	0.93	2.51	3.418 (5)	167

Symmetry codes: (ii) −*x*+5/2, *y*−1/2, −*z*+3/2; (iii) *x*, *y*−1, *z*.
